# Medical-Grade Honey as a Potential New Therapy for Bacterial Vaginosis

**DOI:** 10.3390/antibiotics13040368

**Published:** 2024-04-17

**Authors:** Céline M. J. G. Lardenoije, Senna J. J. M. van Riel, Linsey J. F. Peters, Martine M. L. H. Wassen, Niels A. J. Cremers

**Affiliations:** 1 Department of Gynecology and Obstetrics, Maastricht University Medical Centre+, P. Debyelaan 25, 6229 HX Maastricht, The Netherlands; celine.lardenoije@mumc.nl (C.M.J.G.L.); sjjmvanriel@gmail.com (S.J.J.M.v.R.); 2 Department of Obstetrics & Gynecology, Zuyderland Medical Centre Heerlen, Henri Dunantstraat 5, 6419 PC Heerlen, The Netherlands; m.wassen@zuyderland.nl; 3 VieCuri Medical Centre, Tegelseweg 210, 5912 BL Venlo, The Netherlands; 4 GROW Research Institute for Oncology and Reproduction, Universiteitssingel 40, 6229 ER Maastricht, The Netherlands; 5 Triticum Exploitatie BV, Sleperweg 44, 6222 NK Maastricht, The Netherlands; research@mesitran.com

**Keywords:** bacterial vaginosis, medical-grade honey, vaginal microbiome, lactobacilli, antibiotics, antimicrobial resistance, biofilms, alternative medicine

## Abstract

The prevalence of bacterial vaginosis (BV) among women of reproductive age is 29%. BV arises from a vaginal imbalance marked by reduced levels of lactic acid-producing lactobacilli and an overgrowth of pathogenic anaerobes. The multifactorial nature of BV’s pathogenesis complicates its treatment. Current antibiotic therapy exhibits a recurrence rate of about 60% within a year. Recurrence can be caused by antibiotic treatment failure (e.g., due to antimicrobial resistance), the persistence of residual infections (e.g., due to biofilm formation), and re-infection. Because of the high recurrence rates, alternative therapies are required. Medical-grade honey (MGH), known for its antimicrobial and wound healing properties in wound care, emerges as a potential novel therapy for BV. MGH exerts broad-spectrum antimicrobial activity, employing multiple mechanisms to eliminate the risk of resistance. For example, the low pH of MGH and the production of hydrogen peroxide benefit the microbiota and helps restore the natural vaginal balance. This is supported by in vitro studies demonstrating that MGH has an antibacterial effect on several pathogenic bacteria involved in the pathophysiology of BV, while lactobacilli and the vaginal microenvironment can be positively affected. In contrast to antibiotics, MGH exerts anti-biofilm activity, affects the microbiome as pre- and probiotic, and modulates the vaginal microenvironment through its anti-inflammatory, anti-oxidative, physicochemical, and immunomodulatory properties. More clinical research is required to confirm the positive effect of MGH on BV and to investigate the long-term cure rate.

## 1. Introduction

Bacterial vaginosis (BV) affects up to 29% of women worldwide and is characterized by a vaginal disbalance, a reduced number of lactobacilli, and an increased number of pathogenic bacteria [1]. This leads to vaginal discharge, an unpleasant “fishy” odor, and a negative impact on the self-esteem and quality of life of the women [2,3,4]. Untreated BV can lead to early spontaneous abortion and increase the susceptibility to sexually transmitted infections (STIs) [5,6,7,8]. The pathogenesis of BV is multifactorial. Factors that impact the risk of developing BV can be divided into four main groups: Intrinsic factors (ethnicity and genetics), sexual activity (sex partner, sexually transmitted infections, condom use), hormones (menstrual cycle, intra-uterine device, estrogen levels, oral contraception), and lifestyle (smoking, diet, sugars, vaginal soap, stress, body mass index, use of antibiotics). These risk factors can be protective or stimulating [9,10,11].

BV is characterized by a vaginal disbalance. Commensal bacteria will compete with lactobacilli to bind to the vaginal epithelium. When the number of lactobacilli decreases, commensal bacteria will thrive under an increased pH, adhere to the epithelium, and turn pathogenic. These bacteria can form a polymicrobial biofilm and destroy the vaginal barrier, disrupting the vaginal physiology, and subsequently leading to vaginal discharge and malodor [2,12].

Currently, the standard treatment for BV is oral or intravaginal administration of antibiotics, typically metronidazole or clindamycin [13]. Recurrence rates are high, with 20–30% recurrence after one month and 58% after 12 months of initial treatment. A long-term cure is often hard to achieve and maintain [3,14,15,16,17,18]. An alternative or complementary therapy is the use of probiotics. Probiotics can compete with pathogenic bacteria to bind to the vaginal epithelium, can stimulate host immunomodulatory properties, and may produce bacteriocin, hydrogen peroxide, and biosurfactants with anti-biofilm properties [19,20]. Although a recent meta-analysis from 2022 showed the potential benefit of probiotics as a complementary or alternative treatment, a previous meta-analysis from 2017 showed no benefit [20,21]. Moreover, the route of administration (oral or intravaginal), specific probiotics, and dosing vary greatly [22]. Most literature suggests the need for more high-quality, large-sample randomized controlled trials (RCTs) with a longer follow-up to determine the efficacy of probiotics [20,21,22].

Given the expectation that treatments in the future will become even less effective, e.g., due to the development of antimicrobial resistance and the persistence of biofilms, novel treatment options are warranted. Medical-grade honey (MGH) is already used in wound care for its antimicrobial and wound healing properties and may be a potent alternative treatment.

## 2. Can MGH Create a Paradigm Shift in the Treatment of BV?

Honey has been used since ancient times for the treatment of wounds and other malignancies [23]. Honey was intravaginally applied and used as a gynecological medicine in history [24]. Over the last three decades, strict standards and regulations have been implemented to guarantee that honey can be safely and effectively used for medical applications [25,26]. This resulted in the commencement of MGH, which is organic honey, confirmed to be free of pollutants (such as herbicides, pesticides, heavy metals, and antibiotics), gamma-irradiated (to remove potential harmful endospores, such as *Clostridium botulinum*), and complying with physicochemical criteria (such as glucose, fructose, water content, and other factors to guarantee its consistency and quality), production and storage standards, and legal and safety regulations (in accordance with ISOs and MDR regulations) [26]. In contrast to MGH, honey purchased directly from the beekeeper is not extensively tested for contaminants and has a risk of containing endospores, while honey from the supermarket is often sterilized with heat, which inactivates the enzymes that contribute to its biological activity [27].

**Figure 1:**
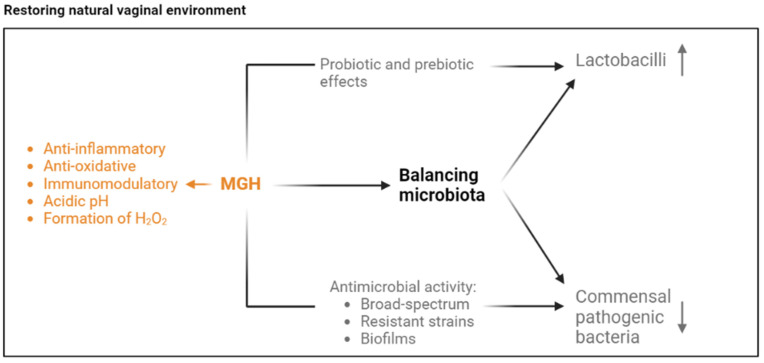
**Proposed mechanisms of how MGH can improve BV pathology.** A disbalance in the vaginal microbiota, such as that experienced during BV, may be improved via multiple mechanisms. Firstly, the acidic pH of honey and the natural formation of low levels of hydrogen peroxide (H_2_O_2_) are part of normal vaginal physiology, and thus this may restore part of the environment. Secondly, honey has anti-inflammatory, anti-oxidative, and immunomodulatory properties that may harness the hostile state of the tissue. Lastly, honey likely improves the vaginal microbiota by its pre- and probiotic effects that may increase the beneficial Lactobacilli and its broad-spectrum antimicrobial activity that decreases the pathogenic bacteria, including antibiotic-resistant strains and those present in biofilms.

MGH exerts multiple mechanisms that may have a positive effect on the vaginal microenvironment and BV pathogenesis (Figure 1). The physiological properties of MGH (acidic pH and presence of hydrogen peroxide) are in line with normal, healthy vaginal physiology [28]. In addition, MGH has anti-inflammatory, anti-oxidative, and immunomodulatory activities that can further help to restore the environment [28]. Moreover, MGH may facilitate balancing the natural vaginal microbiota by its broad-spectrum antimicrobial activity against pathogenic bacteria and its pre- and probiotic activity stimulating lactobacilli [28,29,30,31,32].

## 3. Broad-Spectrum Antimicrobial Activity of MGH

MGH exerts broad-spectrum antimicrobial activity relying on multiple mechanisms: (1) Its osmotic activity, (2) its acidic pH, (3) the formation of hydrogen peroxide, and (4) containing other antimicrobial molecules [33]. Honey consists of more than 200 different components, such as carbohydrates (80–85%), water (15–18%), proteins and amino acids (0.1–0.4%), enzymes, minerals, vitamins, flavonoids, and phenolic compounds [34,35]. The carbohydrates predominantly consist of the monosaccharides fructose and glucose. High-sugar formulations have a strong osmotic activity, attracting water from their surroundings, including from bacteria that depend on water to survive, leading to dehydration and subsequently apoptosis [27,33]. Lactobacilli are better protected against osmotic stress than other bacteria by actively modulating the pool of osmotically active solutes in their cytoplasm, intracellular accumulation of organic compounds, induction of stress proteins, and responding to osmotic signals [36,37,38,39,40]. Honey contains the enzyme glucose oxidase, which is secreted by the bee’s hypopharyngeal glands. Glucose oxidase catabolizes glucose, resulting in the formation of gluconic acid and low amounts of hydrogen peroxide that are toxic for bacteria but not for eukaryotic cells. Most bacteria grow best at a neutral pH, close to 7.0. The pH of honey lies around 3.2–4.5 due to the presence of organic acids, especially gluconic acid, and most microorganisms typically do not foster well in acidic environments [33,41,42]. However, lactobacilli are considered intrinsically resistant to acid [43]. This suggests that honey, with its low pH, can help restore the balance of the vaginal microbiota during BV. Hydrogen peroxide is a strong oxidizing agent that damages lipid membranes and DNA and is cytotoxic to bacteria [27]. Interestingly, some lactobacilli are capable of liberating hydrogen peroxide to the environment as a byproduct of the oxidation of hydrocarbons [44,45]. Moreover, there is a difference in sensitivity to hydrogen peroxide between different bacteria. Bacteria that lack protective mechanisms like catalase or peroxidase are mostly obligate anaerobic bacteria, including Prevotella, and Gardnerella [45,46]. In contrast, lactobacilli possess a mechanism of protection against hydrogen peroxide, based upon the synthesis of hexameric or tetrameric catalase containing manganese, sometimes described as pseudocatalase or catalase/peroxidase [45,47]. Thus, honey can potentially compensate, or supplement, the lost or limited hydrogen peroxide production during BV with low numbers of lactobacilli and help to restore the balance in the vaginal microbiota. Other components, including phenolic compounds (e.g., methylglyoxal (MGO), methyl syringate, and leptosin) and flavonoids (e.g., apigenin, chrysin, pinocembrin, and quercetin), exert additional antibacterial effects [48,49,50]. These effects have been attributed to different mechanisms, including cytoplasmic membrane damage, inhibition of cell wall, and cell membrane synthesis, inhibition of nucleic acid synthesis, and inhibition of energy metabolism [51,52].

The combination of these antimicrobial mechanisms makes MGH highly effective against a broad spectrum of pathogens, irrespective of their resistance profiles, including multi-resistant bacteria (e.g., methicillin-resistant *Staphylococcus aureus* (MRSA), vancomycin-resistant enterococci (VRE), and *Pseudomonas aeruginosa*), fungi (e.g., *Candida albicans*, non-albicans Candida species, and *Candida auris*), and viruses (e.g., Influenza A virus, human immunodeficiency virus (HIV), and herpes simplex virus, type 1 (HSV-1)) [29,30,53,54,55].

Since the antimicrobial activity of MGH is based on multiple mechanisms, microorganisms are unable to develop resistance towards MGH. This is in contrast to antibiotics and antimycotics, in which the antibacterial and antifungal mechanisms are mostly based on one mechanism. Alexander Fleming, the discoverer of penicillin, already warned about the risk of antibiotic resistance in his Nobel Prize laureate in 1945 [56]. Still, antimicrobial resistance is a huge problem worldwide, and the WHO and CDC have flagged it as one of the worst global public health threats facing humanity [57,58,59]. It is important to strictly control the use of antibiotics and find alternatives. Unfortunately, the development of new antimicrobial agents by pharmaceutical companies is limited, likely because they anticipate resistance will eventually develop, as happened in the past, and it is not profitable [42,60,61]. More energy needs to be put into exploring the potency of MGH as an alternative to antibiotics.

Another important difference between antibiotics and MGH is that the latter can also eradicate biofilms, especially when supplemented with vitamins (e.g., L-Mesitran products) [62,63]. Biofilms are aggregated microbes that produce an extracellular matrix (consisting of polysaccharides, proteins, and extracellular DNA), making it vastly difficult for antibiotics and the immune system to reach the microbes and kill them [64,65,66]. Biofilms can be composed of single species or be polymicrobial, and they are up to 1000 times more tolerant to antimicrobial agents and disinfectants than planktonic cells [67,68].

Approximately 80% of chronic and recurrent microbial infections in the human body are due to biofilms [66,69]. In a study with 60 women, a biofilm was present in 90% of women with BV (18 out of 20), while the prevalence was 10% in women without BV (1 out of 20 of premenopausal and 3 out of 20 of postmenopausal women) [70]. The biofilms consisted primarily of *Gardnerella vaginalis*, while *Atopobium vaginae* was present in 80% of the cases [70]. Due to the different physiological activities of MGH, such as its osmotic and debridement activity, and the presence of proteases and other enzymes, biofilms can be penetrated and destroyed [27,71]. Also, MGH reduces the production of extracellular polysaccharide matrix, which leads to the prevention of biofilm formation [28,72].

In addition to its antimicrobial activity in resolving infections, MGH has also shown prophylactic activity when applied locally, typically in wound care, but also in non-conventional indications [72,73,74,75]. Thus, MGH therapy may also be an interesting strategy for the prevention of BV.

Moreover, the in vitro examination of the antibacterial activity of MGH in combination with antibiotics showed that the combination was more effective than the separate therapies [72,76]. The addition of honey reduces the required doses of antibiotics to inhibit bacterial growth, and this can also restore the efficacy of antibiotics in cases of developed antibiotic resistance [72,76,77,78]. These findings suggest that MGH can also have an important role as a complementary therapy to antibiotics. Likely, they have a different effect on the microbes; e.g., antibiotics can enter the bacterial cell more easily after MGH permeabilizes the cell membrane and subsequently interferes with DNA replication and cell growth. Many different molecular mechanisms of how MGH affects bacterial cells are described [42].

## 4. In Contrast to Antibiotics, MGH Acts via Multiple Molecular Mechanisms on Bacterial Cells

Microorganisms are very versatile and adaptive; they can have natural or acquired resistance mechanisms [79]. Natural mechanisms can be intrinsic, in which bacteria are not sensitive to a certain antibiotic by nature, or induced, e.g., due to reduced permeability of the outer membrane or the natural activity of efflux pumps. Acquired resistance follows horizontal gene transfer or mutations to their chromosomal DNA [79]. Antimicrobial resistance can be orchestrated by limiting the uptake of a drug, modifying the target of a drug, inactivating the drug, or actively pumping the drug outside the cell [79].

**Figure 2:**
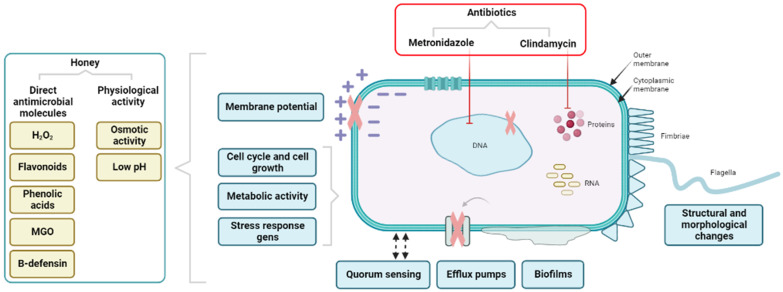
**Differential antimicrobial mechanisms of antibiotics and MGH.** In contrast to antibiotics with one specific antimicrobial mechanism, MGH has multiple mechanisms by which it exerts its broad-spectrum antimicrobial activity. Metronidazole interferes with bacterial DNA replication, while clindamycin inhibits its protein synthesis. MGH contains molecules with direct antimicrobial activity and acts by physiological activity, thereby making it effective against a broad range of microorganisms. These activities together make structural and morphological changes, alter the membrane potential, affect the cell cycle and cell growth, disrupt cell metabolism, influence efflux pump activity, alter quorum sensing (intercellular communication), reduce biofilms, and affect stress responses. Bacteria cannot protect themselves against this multitude of mechanisms and therefore do not develop resistance towards MGH. H_2_O_2_: hydrogen peroxide; MGO: methylglyoxal.

Antimicrobial agents typically target one mechanism to decrease the pathogenicity of microbes, accounting for evolutionary pressure on the bacteria because they are able to adapt easily [42,80]. The antibiotics used to treat BV are based on a single mechanism (Figure 2). Metronidazole interferes with DNA replication in anaerobic bacteria by causing the formation of toxic metabolites after the reduction of the nitro-groups of the drug, thereby stopping their growth [81,82]. Different mechanisms may induce resistance towards metronidazole, including reduced uptake by efflux or altering the reductive pathways, inactivating the toxic metabolites, and increasing DNA repair mechanisms [82,83]. Clindamycin inhibits bacterial protein synthesis by binding to the bacterial ribosome, interfering with the assembly of the ribosome and its translation into proteins [84,85]. Resistance to clindamycin may be induced by modification of the target binding site or active efflux from the bacterial cell [85].

In contrast to antibiotics, it has been described that MGH kills bacteria by targeting multiple molecular aspects, making MGH highly effective against a broad spectrum of microorganisms (Figure 2) [42]. Since the antimicrobial activity of MGH is based on multiple mechanisms, microorganisms are unable to develop resistance towards MGH [29,80]. The molecular mechanisms by which MGH affects the microbes have recently been extensively reviewed by Combarros-Fuertes et al. and include: Making structural and morphological changes, altering the membrane potential, affecting cell cycle and cell growth, disrupting cell metabolism, influencing efflux pump activity, altering quorum sensing (intercellular communication), reducing biofilms, and affecting stress responses [42,80,86,87]. Together, they will strongly limit the virulence of pathogens. Interestingly, different microorganisms tend to be differentially susceptible to MGH, and some beneficial bacteria, such as lactobacilli, may be limitedly affected, possibly as a result of different sensitivities to osmotic pressure, the low pH, and the hydrogen peroxide generated by MGH. Table 1 summarizes the activity of MGH against the different bacterial species involved in BV.

## 5. MGH Likely Has a Positive Effect on the Vaginal Microbiota

The collection of microorganisms that resides in a specific part of the body, such as the skin, gut, or vagina, is called the microbiota. The microbiota plays an essential role in maintaining the function of the specific organ; e.g., the gut microbiota helps in the digestion and absorption of nutrients, synthesizes vitamins, and strengthens the immune system. A disbalance in the vaginal microbiota can lead to complaints, e.g., as is the case in BV. Antibiotics can kill specific pathogenic bacteria; however, they may also kill other commensal bacteria, which further disbalances the microbiota. Treatment with prebiotics and probiotics can help restore the normal microflora by providing or stimulating the growth of healthy microorganisms. Honey may be a potent option to regain a healthy, balanced microbiota.

It is described that honey consumption can have beneficial activities on the gut microbiota by reducing the infection-causing bacteria, such as *Salmonella*, *Escherichia coli*, and *Clostridiodes difficile,* while stimulating the growth of potentially beneficial species, such as lactobacilli and bifidobacteria [31,32]. These latter two are most often used as probiotics [100,101]. Interestingly, they also exist in the hindgut of the bees, with the lactic acid bacteria species being the most dominant [102]. The diet of honey bees is fructose rich, and fructophilic bacteria, e.g., certain lactic acid bacteria, can survive in both the gastrointestinal tracts of these insects and honey [103]. Food containing probiotics is popular around the world because of their potential health advantages [100]. Honey may contain probiotics that have been transmitted from the guts of bees during the process of making honey and may remain alive for a certain period [104]. Besides, when medical-grade clover honey is supplemented with an infant’s milk formula, it can also have prebiotic activity, decreasing colonization with Entereobacteriaceae and increasing *Bifidobacterium bifidum* and lactobacilli [105]. Moreover, the oligosaccharides in honey can promote the growth of bifidobacteria and lactobacilli [32].

Thus, honey may affect the vaginal microbiota through its prebiotic and probiotic activity, which stimulates the growth of beneficial bacteria, and through its antimicrobial activity, which decreases colonization with pathogenic bacteria. Local application of MGH may skew the balance in the right direction. Decreasing the pathogenic bacterial load will lead to clinical benefits by reducing complaints, such as reducing malodor. One of the things that stands out in wound care is that MGH reduces malodor within a couple of days after initiating MGH treatment. This has been attributed to honey providing carbohydrates as an alternative nutrient source for the bacteria, instead of catabolizing tissue debris and proteins, consisting of amino acids containing sulphur and nitrogen groups (associated with bad smells) [106]. Similarly, in BV, it is known that the malodor is caused by the decarboxylation of amino acids by bacteria into biogenic amines, such as putrescine and cadaverine [107,108,109]. Thus, MGH can likely also reduce the fishy odor in BV patients. Honey also has antifungal activity, and clinical studies have shown that honey has a positive effect on treating vaginal fungal infections. Therefore, honey may also have an impact on commensal fungi and, subsequently, the balance of the vaginal microbiota. This should be explored in future clinical studies that investigate the effect of MGH on the treatment of BV.

## 6. MGH Exerts Anti-Inflammatory, Anti-Oxidant, and Immunomodulatory Activity

The micro-environment of the vagina can get into a pro-inflammatory state due to an imbalance between the beneficial lactobacilli and pathogenic bacteria [110,111,112]. Inflammation is less pronounced in localized BV and is mostly observed during persistent BV [113]. However, excessive levels of pathogenic bacteria cause cellular stress with elevated pro-inflammatory cytokines along with reactive oxygen species (ROS) that trigger an immune response, which subsequently instigates vaginal inflammation [110,111,114]. MGH can influence these processes by its anti-inflammatory, anti-oxidative, and immunomodulatory activities [73].

MGH contains several phytochemicals and bioactive molecules, such as flavonoids, polyphenolic compounds, glycoproteins, glycopeptides, and vitamin C [115,116]. These molecules can scavenge ROS, reducing inflammation and minimizing tissue damage [117,118,119]. MGH harnesses the tissue for new infections by attenuating the inflammatory state and skewing it towards protection. The same bioactive compounds can also have immunomodulatory activities and modulate the immune system’s response by stimulating or suppressing certain immune cells, including T cells, B cells, natural killer cells, and macrophages. They can also regulate the production of pro- and anti-inflammatory cytokines, which play a crucial role in the immune response. Leukocytes migrate in response to cytokines and chemokines that are produced under the influence of the local microenvironment [120,121]. By influencing the microenvironment (pH, hydrogen peroxide, inflammatory, and oxidative state), MGH can modulate immunological mediators and affect the immune response [122]. MGH may treat and prevent infections by modulating immune cell activities and cytokine production, either directly or indirectly, through its antimicrobial activity that affects bacterial colonization and thus prevents a subsequent immune response to these pathogens [28,123]. MGH does also prevent infections, and its prophylactic activity has been demonstrated previously in different settings (e.g., lacerations, colic surgery, caesarean section, and heel pressure ulcers) [124,125,126,127].

## 7. Discussion of the Clinical Evidence of the Concept of MGH for Treating BV

Although the clinical evidence of MGH for the treatment of BV is scarce, the concept of using MGH is interesting and should be investigated in more detail. Traditionally, natural compounds, including plant extracts, essential oils, and antimicrobial peptides, have been used to treat vaginal dysbiosis [128]. Some of these products, e.g., *Thymbra capitata* essential oil, are effective against BV-associated bacteria [128]. Interestingly, these products or MGH can be used in combination with antibiotics or probiotics to improve their efficacy and reduce recurrence. Local, intra-vaginal application may be the most effective.

To our awareness, there is only one study that investigates the use of honey for the treatment of BV. In a pilot study with thirty patients with vaginal complaints, an MGH-based product (L-Mesitran ointment) was applied daily for at least one week [129]. All patients had either excessive or crumbly secretion, in combination with symptoms such as painful sex (n = 14), itching (n = 3), or a burning sensation (n = 15). Microscopic evaluation of the smears determined 12 patients had BV, 11 had candidiasis, 4 had lactobacillosis, 1 had trichomonas, and 2 of the patients had a normal flora. Almost all women found the effect of MGH treatment to be noticeable (n = 27), while two were helped a little bit, with less painful sex mentioned in particular. Also, microscopic evaluation of the smears 7 days after starting treatment demonstrated a positive effect in most cases [129].

Two clinical trials investigated the use of honey for treating cervicitis. Cervicitis is an irritation or infection of the cervix and can be caused by STIs or bacterial vaginosis. In the first study, 97 patients were included, of which 72 returned for follow-up [130]. All parameters, including vaginal irritation, itching, discharge, bleeding, and pain after coitus, tenderness, and cervical wound size and friability, improved significantly in comparison to the time before starting treatment with honey. Five patients (6.9%) had vulvovaginal itching after treatment that resolved after 3–4 days. They concluded that daily vaginal administration of flixweed–honey using applicators for two weeks could be used in the treatment of clinical symptoms of cervicitis, irrespective of the cause [130]. In the second study, a double-blind RCT with 102 women investigated the adjuvant therapy with Ziziphus honey compared to standard oral antibiotic treatment [131]. Participants applied 5 mL of honey with an applicator every night for two weeks. All clinical symptoms of cervicitis significantly decreased after the treatment in both groups (*p* < 0.05), but the vaginal discharge in the adjuvant treatment group reduced significantly more (96% vs. 64.7%, *p* < 0.001). In addition, the restoration of cervical erosion in a subpopulation of the patients was significantly better when compared with the control group (100% vs. 44%, *p* = 0.02). They concluded that vaginal honey adjuvant therapy can improve the efficiency of standard antibiotic treatment to reduce the clinical symptoms of cervicitis [131].

In an in vivo BV model in rhesus macaques, application of 0.5 g of 9% sucrose gel (one of the carbohydrates in honey) with a syringe to the fornix of the vagina for five consecutive days reduced the vaginal pH and induced a shift in the vaginal flora from a BV phenotype to lactobacilli-dominated flora [132]. In another in vivo study with Sprague-Dawley rats, the effect of gelam honey, which has strong anti-inflammatory and anti-oxidative activities, on the uterine and vaginal epithelial thickness was investigated [133]. Honey was daily administered orally for 14 days in 0.5 mL doses with a concentration of 0.2–8 g honey per kg body weight per day, with water as a vehicle. Honey attenuated the atrophy of uterine and vaginal epithelia and increased the thickness of the endometrial stroma and endometrial surface endothelial layer [133]. The vaginal mucosal barrier plays a pivotal role in BV pathogenesis, and therefore the thickening by honey is a positive outcome [128].

The clinical evidence for MGH as an alternative to antibiotics for the treatment of BV is currently limited. Therefore, well-performed clinical studies are important and encouraged to further investigate the potential of MGH for the treatment of BV. Not all honey is MGH, and in many cases, such as in the here presented in vitro and (pre)clinical studies, especially when used as traditional medicine, regular honey is used for medical applications. However, in modern medicine, it makes more sense to use standardized, high-quality honey for medical applications to guarantee its safety and its consistent efficacy, urging the use of MGH. MGH has multiple properties that make it an interesting complementary or alternative treatment.

## 8. Conclusions

BV is a large problem among women, negatively affecting their quality of life. Current therapies with antibiotics frequently lead to the recurrence of BV. MGH may possibly be a potent alternative or complementary therapy to antibiotics. MGH exerts broad-spectrum antimicrobial and anti-biofilm activity and can restore the vaginal microbiota by promoting lactobacilli and decreasing pathogenic bacteria as a pre- and probiotic. In addition, the combination of its low pH, the production of hydrogen peroxide, and its anti-inflammatory, anti-oxidant, and immunomodulatory activities may further promote an optimal vaginal environment without complaints. However, clinical studies are needed to confirm the conceptually positive effect of MGH on BV and to investigate the long-term cure rate.

## Figures and Tables

**Table 1:** The in vitro effect of honey on Lactobacilli and facultative pathogenic bacterial species relevant to the vaginal microbiota. % *w*/*v* = percent weight/volume (g/mL); green marking indicates a positive effect; red marking indicates a negative effect regarding its survival or growth; white marking indicates no effect.

Bacterial Strain	Honey	References
**Lactobacilli**		
Lactobacilli	Feeding 2 g/day of local Indian honey to rats increased the counts of lactic acid bacteria in their intestines	[88]
*L. acidophilus* (strong probiotic; commonly found in the gastrointestinal tract, oral cavity, and vagina)	Local Indian honey (1% sugar) increased bacterial count in vitro 3% *w*/*v* pasteurized Brazilian honey increased the number of viable Lactobacilli in milk 46 days after storage Unknown % honey (light, amber, and dark) increased bacterial count in ice cream 5% *w*/*v* honey (Dabur, India) showed an increased bacterial count in milk	[88,89,90,91,92]
	5% *w*/*v* clover honey showed no effect on bacterial count in milk	
*L. casei var. rhamnosus * (probiotic vagina)	5% *w*/*v* honey (Dabur, India) increased bacterial count in milk	[91]
*L. plantarum * (probiotic bowel)	5% *w*/*v* honey (Dabur, India) increased bacterial count in milk	[91]
*L. delbrukeii* subsp. *bulgaricus* (yoghurt production)	5% *w*/*v* clover honey did not affect the bacterial count in milk	[92]
**Facultative pathogenic bacteria**		
*G. vaginalis*	7% *w*/*v*, formulation, manuka honey decreased the number of bacteria	[93]
*Atopobium vaginae*	7% *w*/*v*, formulation, manuka honey decreased the number of bacteria	[93]
*Ureaplasma parvum*	20% *w*/*v*, manuka honey decreased the number of bacteria	[94]
*Ureaplasma urealyticum*	20% *w*/*v*, manuka honey decreased the number of bacteria	[94]
*Prevotella intermedia*	50% *w*/*v*, different honey types decreased the number of bacteria	[95,96]
*Pophyromonas gingivitis*	Manuka, MGO, and hydrogen peroxide decreased the number of bacteria	[97]
*Fusobacterium* spp.	6.3–25% *w*/*v* Manuka, 25% *w*/*v* citrus honey, 5.9 ± 0.9% (*w*/*v*) *Saturja* spp. honey, 6.25% *w*/*v* oregano and sage honey decreased the number of bacteria	[98]
*Clostridiales* spp.	*Clostridium difficile*: 6.25% *v*/*v* MIC (Manuka)	[99]
*Bacteroides* species/ *P. oris*	Different undiluted honey types tested in disc diffusion assay: 8.9–12.1 mm zone of inhibition *Bacteroides* spp. and 8.5–14.9 mm zone of inhibition *P. oris*	[96]

## Data Availability

No new data were created or analyzed in this study. Data sharing is not applicable to this article.
